# Valproate induced encephalopathy: Paradigm of normal ammonia levels

**DOI:** 10.1192/j.eurpsy.2021.1642

**Published:** 2021-08-13

**Authors:** P. Barbosa, O. Nombora, J. Monteiro, L. Ribeiro

**Affiliations:** Psychiatric Department, Vila Nova de Gaia/Espinho Hospital Center, Vila Nova de Gaia, Portugal

**Keywords:** Normal Ammonia levels, Valproate, Mood stabilizer, Encephalopathy

## Abstract

**Introduction:**

Valproic Acid (VPA) is one of the most commonly used mood stabilizer drugs. Although uncommon, serious adverse effects have been reported. One particularly relevant side effect is the induced encephalopathy, usually secondary to Hyperammonemia. However, some descriptions have shown an altered mental state with normal serum levels of ammonia.

**Objectives:**

We aim to present a case of VPA induced-encephalopathy without hyperammonemia and emphasize its suspicion when patients taking VPA present altered mental states.

**Methods:**

We present a clinical case of VPA induced-encephalopathy without Hyperammonemia and a qualitative review of this topic using the Pubmed database.

**Results:**

A 66-year-old woman, with an history of Major Depressive Disorder, previously medicated with Venlafaxine 75mg/day and Mirtazapine 30mg/day, was admitted in our acute psychiatric inpatient unit due to a first manic episode. During the stay, her antidepressants were interrupted, and she was started on VPA, then optimized to 750mg/day. After that, she presented an altered mental state with confusion and prostration. Analytical results were normal including normal ammonia levels and no imagiological abnormalities. Despite these results, we decided to stop VPA empirically. The patient clinical status resolved the day after.

**Conclusions:**

Studies have shown that only a few patients have developed encephalopathy with normal serum levels of ammonia. Although the pathogenesis behind this remains unknown, a few mechanisms have been proposed. Therefore, it is important to remind that even without abnormal analytical status, VPA is a possible cause of encephalopathy. We also emphasize the need for further studies on the mechanisms behind this phenomenon.

**Disclosure:**

No significant relationships.

